# Ultrasound‐Activated Piezoelectric MoS_2_ Enhances Sonodynamic for Bacterial Killing

**DOI:** 10.1002/smsc.202300022

**Published:** 2023-04-19

**Authors:** Chaofeng Wang, Wenchan Sun, Yiming Xiang, Shuilin Wu, Yufeng Zheng, Yu Zhang, Jie Shen, Lei Yang, Chunyong Liang, Xiangmei Liu

**Affiliations:** ^1^ School of Life Science and Health Engineering Hebei University of Technology Xiping Avenue 5340 Tianjin 300401 China; ^2^ Biomedical Materials Engineering Research Center Hubei Key Laboratory of Polymer Materials Ministry-of-Education Key Laboratory for the Green Preparation and Application of Functional Materials School of Materials Science & Engineering Hubei University Wuhan 430062 China; ^3^ School of Materials Science & Engineering Peking University Yiheyuan Road 5# Beijing 100871 China; ^4^ Department of Orthopedics Guangdong Provincial People's Hospital Guangdong Academy of Medical Sciences Zhongshan 2nd Road 106# Guangzhou 510080 China; ^5^ Shenzhen Key Laboratory of Spine Surgery Department of Spine Surgery Peking University Shenzhen Hospital Lianhua Road 1120#, Futian District Shenzhen 518036 China

**Keywords:** antibacterial, bacterial infection, heterostructure, piezoelectric, sonodynamic

## Abstract

Bacterial infections are a serious public health issue that threatens the lives of patients because of their ability to induce other lethal complications without prompt treatment. Conventional antibiotic therapy can cause bacterial resistance and other adverse effects. It is highly desirable to develop effective and antibiotic‐independent therapeutic strategies to treat various kinds of bacterial infections. Herein, sonodynamic‐enhanced piezoelectric materials MoS_2_ and Cu_2_Oheterostructure that responds to exogenous ultrasound (US) and generates reactive oxygen for *Staphylococcus aureus* elimination are developed. It is shown in the results that the polariton charge induced by piezoelectric MoS_2_ nanosheets under US irradiation can accelerate the transfer of electric in Cu_2_O. Furthermore, US irradiation induces valence conversion from Cu(I) to Cu(II), which can accelerate glutathione oxidation significantly and destroy the bacterial antioxidant defense system. Hence, the as‐prepared piezoelectric‐enhanced sonosensitizer possesses a highly effective antibacterial efficacy of 99.85% against *S. aureus* under US irradiation for 20 min, with good biocompatibility. Herein, effective ultrasonic piezocatalytic therapy is offered through constructing heterogeneous interfaces with ultrasonic piezoelectric response.

## Graphical abstract

MoS_2_/Cu_2_O (MC) heterojunctions are synthesized by hydrothermal and in situ growth methods. The antibacterial mechanism of MC is studied. MoS_2_ has piezoelectric properties and can be polarized by ultrasonic excitation, and the polarized charge generated can effectively transfer electrons in Cu_2_O. Based on the reactive oxygen species generated and depletion of glutathione, the antibacterial rate of MC can reach 99.85%.

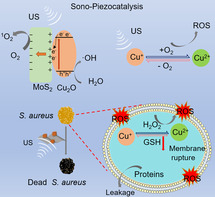

## 1 Introduction

Bacterial infections pose a great threat and burden to human health.^[^ ^1^ ^]^ Each year, a large number of people die from pneumonia, sepsis, and other bacterial infections.^[^ ^2^, ^3^, ^4^ ^]^ *Staphylococcus aureus* is a common pathogen of skin wound infections that often develop complications, such as bacterial pneumonia, osteomyelitis, and so on.^[^ ^5^ ^]^ Therefore, it is crucial for human health to quickly and efficiently eradicate these dangerous germs. Currently, antibiotic treatment has become the most commonly used method to combat bacterial illnesses in the clinical setting.^[^ ^6^ ^]^ However, resistance to bacteria due to the overuse of antibiotics poses a greater threat to human health.^[^ ^7^, ^8^ ^]^ Therefore, there is an urgent need to develop new antibiotic‐free methods for rapid sterilization.

Photodynamic and photothermal methods to eliminate bacteria have attracted extensive attention in recent years, due to reactive oxygen species (ROS) and hyperthermia being produced by various kinds of photoresponsive materials under light stimulation.^[^ ^9^, ^10^, ^11^ ^]^ Unfortunately, because of the limited penetration ability of near‐infrared light, it cannot be used to treat deep tissue infections, which greatly limits the application scenarios of photocatalytic therapy.^[^ ^12^ ^]^ Compared to photodynamic therapy, sonodynamic therapy (SDT) can use ultrasonic waves to excite sonosensitizers to ROS and oxidative stress to treat deeper tissue infections.^[^ ^13^ ^]^ In particular, ultrasound (US)‐responsive materials are currently mainly derived from the photosensitizer metalloporphyrin, noble‐metal carbon materials, inorganic materials (TiO_2_), curcumin with nanostructures, carbon materials, and piezoelectric nanoparticles.^[^ ^14^, ^15^, ^16^, ^17^, ^18^ ^]^ Similar to photosensitizers, the catalytic performance of sonosensitizers depends on the electron‐transfer efficiency of ultrasonic excitation.^[^ ^19^ ^]^ Further modification of the sonosensitizer material is the most effective way to improve its catalytic performance. Currently, the main methods are the use of noble metal materials to modify the sonosensitizer; while improving the catalytic performance, its toxicity is also greatly increased. Therefore, it is important to develop a good biocompatible sonocatalytic material. With their unique piezoelectric effects, piezoelectric materials, such as barium titanate (BaTiO_3_),^[^ ^20^ ^]^ MoS_2_ nanosheets,^[^ ^21^ ^]^ and black phosphorus, can instantly create a built‐in electric field when subjected to external mechanical stress.^[^ ^22^ ^]^ Under ultrasonic excitation, a piezoelectric material is polarized, and electrons gather on one side but quickly neutralize the surrounding charge, which greatly limits their catalytic performance.^[^ ^23^ ^]^ In particular, when piezoelectric materials and semiconductor materials are combined to form a heterojunction, the polarized electrons excited by ultrasound at the interface are efficiently migrated; thus, the yield of electrons can be increased to produce more ROS.^[^ ^24^ ^]^

Cu(I), as a low‐valent transition‐metal cation, has the properties of a Fenton/Fenton‐like agent that catalyzes the production of ROS from hydrogen peroxide.^[^ ^25^, ^26^ ^]^ Importantly, bacterial endogenous glutathione (GSH) can regulate the transition of the copper valence state and destroy the internal structure of bacteria.^[^ ^27^, ^28^ ^]^ However, these effects are far less than the requirements for the killing of bacteria. Therefore, the adoption of external energy, such as US excitation, the regulation of the reversible conversion of Cu(I) and Cu(II) valence states, and the depletion of GSH inside bacteria are potential treatment strategies to achieve the requirements of a rapid and precise treatment of bacterial infections.^[^ ^29^ ^]^ Cuprous oxide (Cu_2_O), rich in large amounts of Cu(I), is a semiconductor with a narrow bandgap and is widely used in various catalytic reactions.^[^ ^30^, ^31^, ^32^ ^]^ In addition, based on the US induced between separation electrons and holes, Cu_2_O are employed as sonosensitizers converting H_2_O and O_2_ into ROS for SDT.^[^ ^33^, ^34^, ^35^ ^]^

**Scheme 1:**
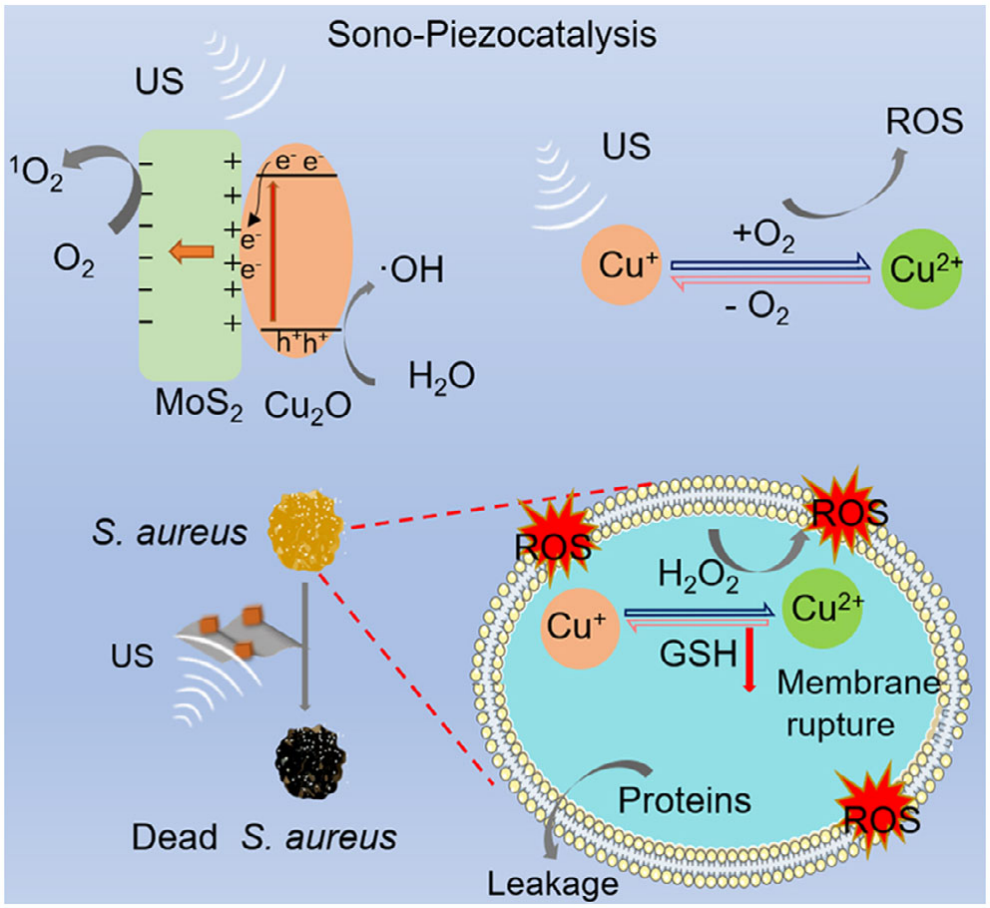
Sono‐piezocatalysis mechanism of antibacterial.

Herein, we created a piezoelectric‐assisted, valence‐adjustable sonosensitizer heterojunction structure with a strong US‐responding ability to treat *S. aureus* infection through efficient SDT. Based on the previous analysis, the Cu_2_O nanocube was chosen as a sonosensitizer because it has not only good electrical conductivity but also a valence structure with ultrasonic excitation. MoS_2_ and Cu_2_O heterojunctions were synthesized using a simple hydrothermal method. MoS_2_ nanosheets with piezoelectric effects were modified on the surface of Cu_2_O, which could augment the antibacterial SDT ability of Cu_2_O through a sono‐piezoelectric polarization effect and mechanical force. On the one hand, in the construction of heterojunctions, the electrons generated by piezoelectricity at the interface of heterojunctions are rapidly transferred so that their ultrasonic catalytic effect is enhanced. On the other hand, the US regulation of the conversion of Cu(I) and Cu(II) can rapidly oxidize GSH inside bacteria and further improve antibacterial efficiency. Consequently, the as‐prepared MoS_2_/Cu_2_O (MC) exhibited highly effective bacterial killing efficacy due to the sonocatalytic, as well as piezocatalytic, properties, as schematically illustrated in **Scheme** **1**.

## 2 Results and Discussion

### 2.1 Characterization of Morphology and Structure Materials

**Figure 1:**
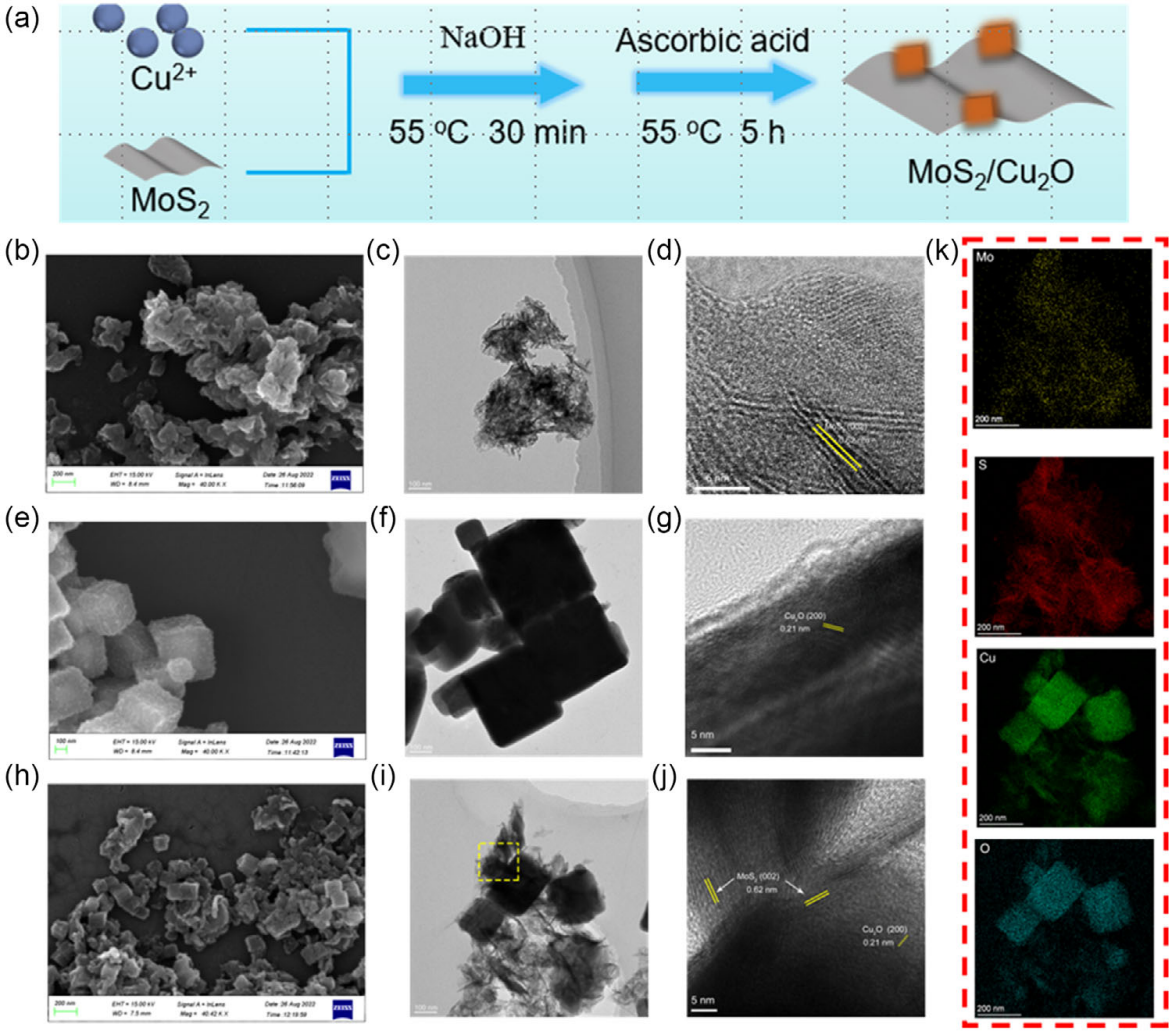
Synthesis and characterization of MoS_2_@Cu_2_O (MC) heterostructure, a) Schematic illustration of the synthetic procedure of MC nanoparticle, b) the field emission scanning electron microscope (FE‐SEM) image of MoS_2_, c) transmission electron microscope (TEM) image of MoS_2_, d) high‐resolution TEM (HRTEM) of MoS_2_, e) the FE‐SEM image of Cu_2_O, f) TEM image of Cu_2_O, g) HRTEM of Cu_2_O, h) the FE‐SEM image of MC, i) TEM image of MC, j) HRTEM of MC, and k) elemental mapping images of MC.

**Figure 2:**
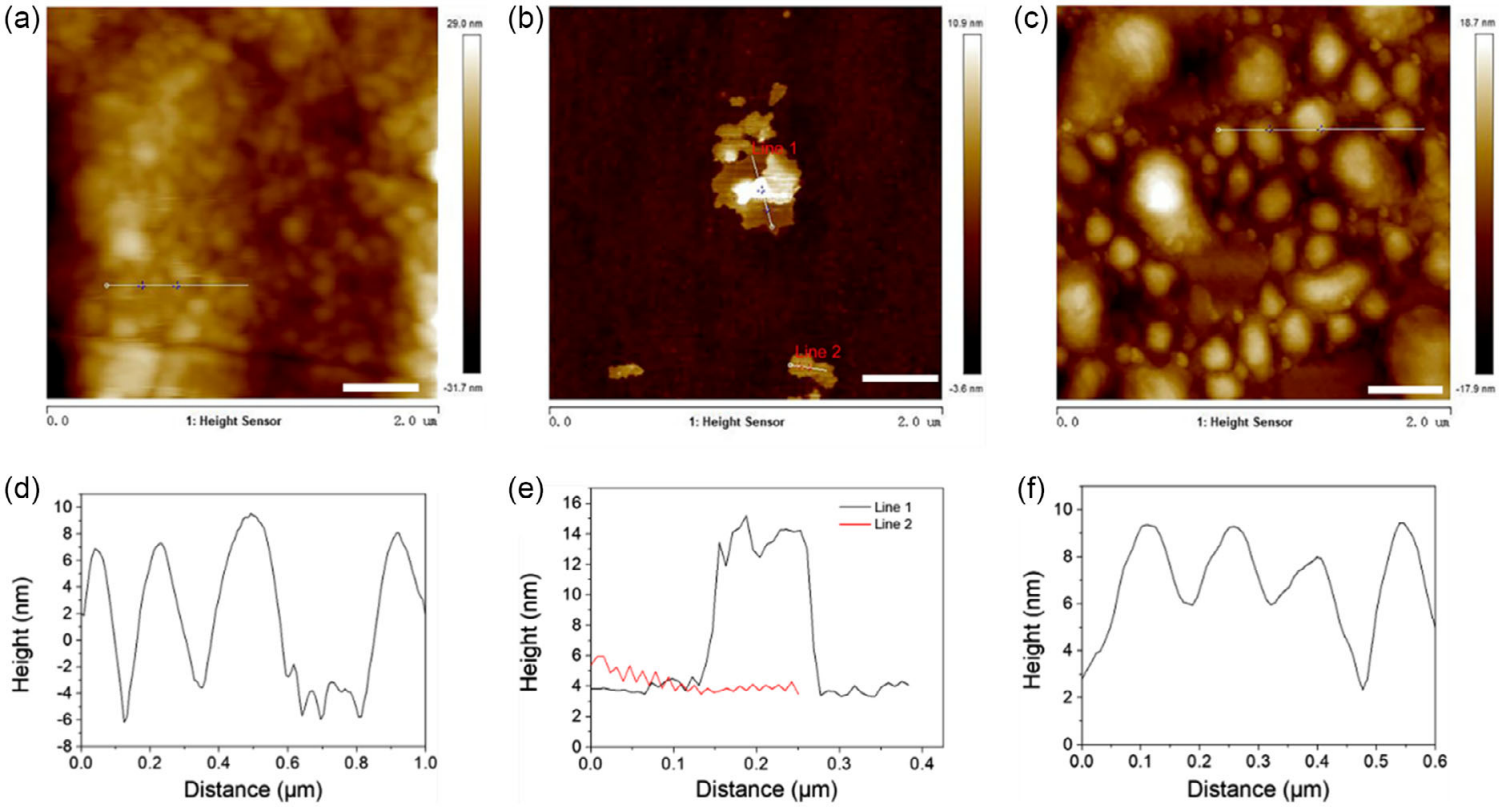
Atomic force microscope (AFM) image (scale bar = 400 nm) of a) Cu_2_O, b) MoS_2_, c) MC, and corresponding height profiles of d) Cu_2_O, e) MoS_2_, and f) MC.

A schematic of the preparation process for MC is presented in **Figure** **1**a. The morphologies of the materials (MoS_2_, Cu_2_O, MC) were observed by field‐emission scanning electron microscopy (FE‐SEM). The SEM image of MoS_2_ is shown in Figure 1b. The images show that MoS_2_ is composed of nanosheets with abundant active sites. The transmission electron microscopy (TEM) image of MoS_2_ (Figure 1c) also confirmed the observed structure of MoS_2_ is a lamellar. The lattice spacing of 0.62 nm observed by high‐resolution TEM (HRTEM) (Figure 1d) is the (002) crystal plane belonging to MoS_2_.^[^ ^5^, ^11^, ^36^ ^]^ Element mapping (Figure S1a,b, Supporting Information) showed that Mo and S were uniformly distributed on the MoS_2_ nanosheets. The prepared Cu_2_O exhibited a nanocube morphology (Figure 1e), which corresponded to the TEM image of Cu_2_O (Figure 1f). As shown in Figure 1g, the lattice spacing of 0.21 nm was consistent in the (200) crystal plane of Cu_2_O.^[^ ^37^, ^38^ ^]^ Element mapping (Figure S2a,b, Supporting Information) showed that Cu and O were uniformly distributed on the Cu_2_O nanocube. The SEM and TEM images (Figure 1h,i) showed a similar core–shell structure of the synthesized MC (i.e., nanocubes were encapsulated by layer‐like nanosheets). The presence of a heterojunction interface between Cu_2_O and MoS_2_ was observed by HRTEM, and defects due to lattice distortion were also observed in molybdenum sulfide. Energy‐dispersive spectroscopy (EDS) element mapping (Figure 1k) showed that Cu and O were uniformly distributed on the Cu_2_O nanosheets, whereas the elements Mo and S were homogeneously distributed on the plane of MoS_2_. Figure S3 shows the content of each element of MC, indicating that Mo, S, Cu and O are 2.76%, 17.41%, 53.17%, and 26.11%, respectively. Inductively coupled plasma (ICP) detection corresponding to Table 1 (Supporting information) shows that the contents of Mo and Cu are 5.7884% and 59.7277%, respectively. Furthermore, as shown in **Figure** **2**, the atomic force microscope (AFM) image and the corresponding height profiles agreed well with the TEM images of these different samples. Figure 2a,d shows that the Cu_2_O nanocubes were more homogeneous in size and are of nanoscale. As shown in Figure 2e, the AFM image and the corresponding height profiles of MoS_2_ nanosheets demonstrated an average thickness of ≈3.0 nm, which proved the lamellar structure of MoS_2_. The AFM image (Figure 2c) and the corresponding height profiles (Figure 2f) showed that the average thickness of MC was greater than that of MoS_2_ and Cu_2_O, which proved the successful compounding of MoS_2_ nanosheets and Cu_2_O. The 3D section analysis of Cu_2_O, MoS_2_, and MC with AFM image is shown in Figures S4–S6, Supporting Information, respectively.

**Figure 3:**
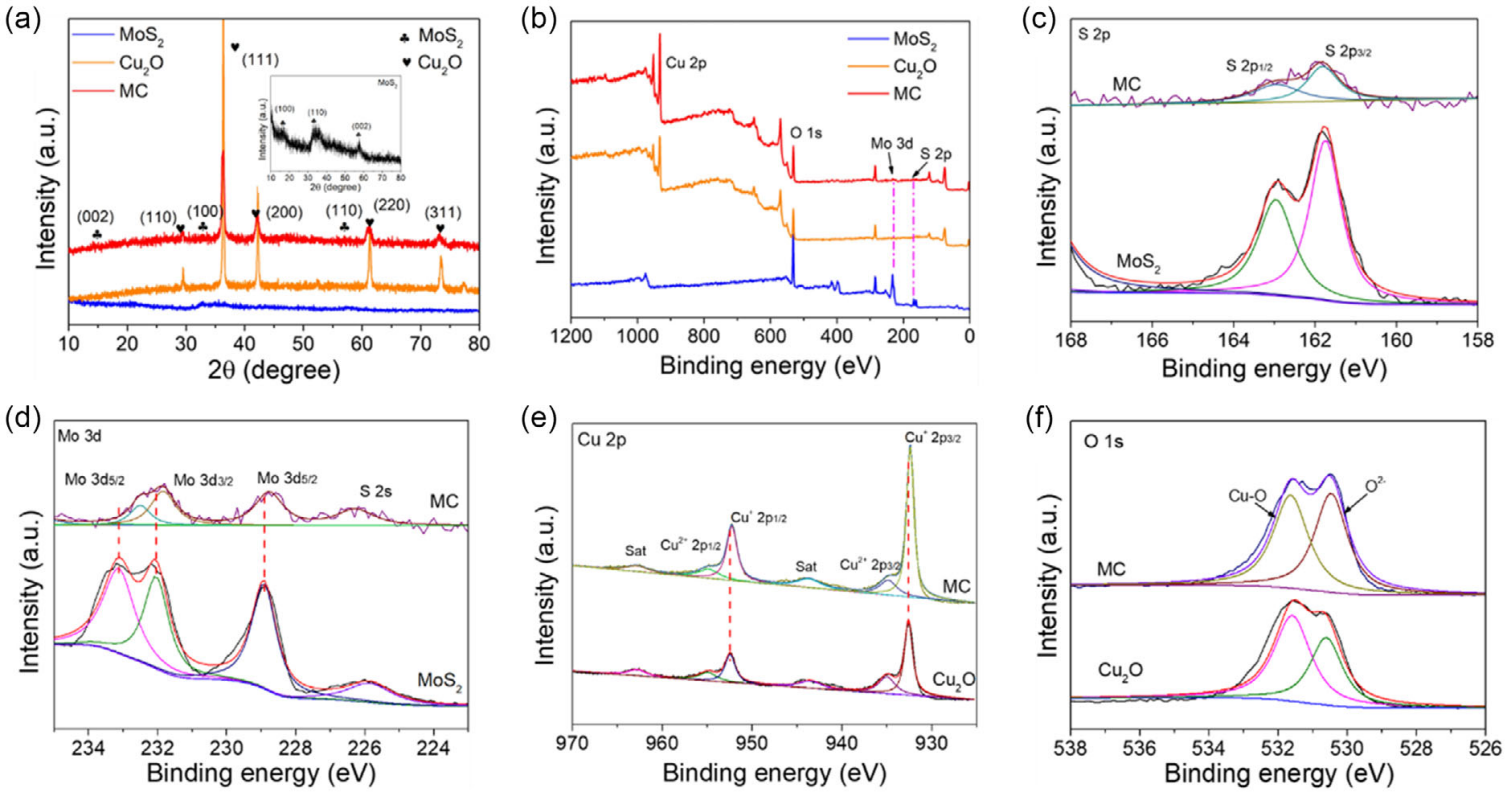
The phase structure and chemical composition of MC. a) X‐ray diffraction (XRD) spectrum of different samples, b) X‐ray photoelectron spectroscopy (XPS) survey spectra, c) high‐resolution XPS spectra of S 2*p* of MoS_2_ and MC, d) high‐resolution XPS spectra of Mo 3*d* of MoS_2_ and MC, e) high‐resolution XPS spectra of Cu of Cu_2_O and MC, and f) high‐resolution XPS spectra of O of Cu_2_O and MC.

**Figure** **3**a shows the X‐ray diffraction (XRD) patterns of the as‐prepared of Cu_2_O, MoS_2_, and MC. The XRD pattern of MoS_2_ exhibited diffraction peaks at 14.3° (002), 33.8° (100), and 58.78° (110), which agreed well with the diffraction patterns of the hexagonal 2H‐phase of MoS_2_.^[^ ^11^, ^36^ ^]^ The diffraction peak of MoS_2_ in MC was weaker than that of Cu_2_O, which may be caused by the relatively small amount of MoS_2_ in MC. To further investigate the combinations on the valence state of elements in the as‐prepared materials, we used the X‐ray photoelectron spectroscopy (XPS) spectra. As shown in Figure 3b, the survey scan of the MC (red curve) indicated the existence of diffraction peaks of Mo, S, Cu, C, and O. Figure 3c shows that the high‐resolution spectrum of S 2*p* obtained from MoS_2_ displayed two peaks at 163.18 (S 2*p*_1/2_) and 161.70 eV (S 2*p*_3/2_).^[^ ^39^ ^]^ The S 2*p* was tested at lower binding energies in the MC than MoS_2_, which was the difference in electronegativity between S and Cu, the electron cloud density around S decreased. The Mo 3*d* XPS spectrum is shown in Figure 3d. The peaks of 228.9 and 232.4 eV belonged to Mo 2*d*_3/2_ and Mo 2*d*_5/2_, respectively, indicating the presence of Mo (IV).^[^ ^40^ ^]^ In contrast, the Mo 3*d* binding energies of MC were redshifted to lower binding energies compared to MoS_2_, indicating an interaction between MoS_2_ and Cu_2_O in the MC hybrid. Bands at 932.46 and 952.36 eV were attributed to Cu(I) (Figure 3e). Moreover, the peaks at 933.93 and 953.96 eV were assigned to Cu^2+^ 2*p*_1/2_ and Cu^2+^ 2*p*_3/2_, respectively. The O 1*s* spectrum in Figure 3f shows that the bands at 530.38 and 531.68 eV belonged to lattice oxygen and Cu_2_O, respectively.^[^ ^36^ ^]^ The XPS spectrum results demonstrate that the MoS_2_ and MC successfully composition, which is consistent with XRD and TEM results.

### 2.2 Investigation of Sonodynamic Effects

**Figure 4:**
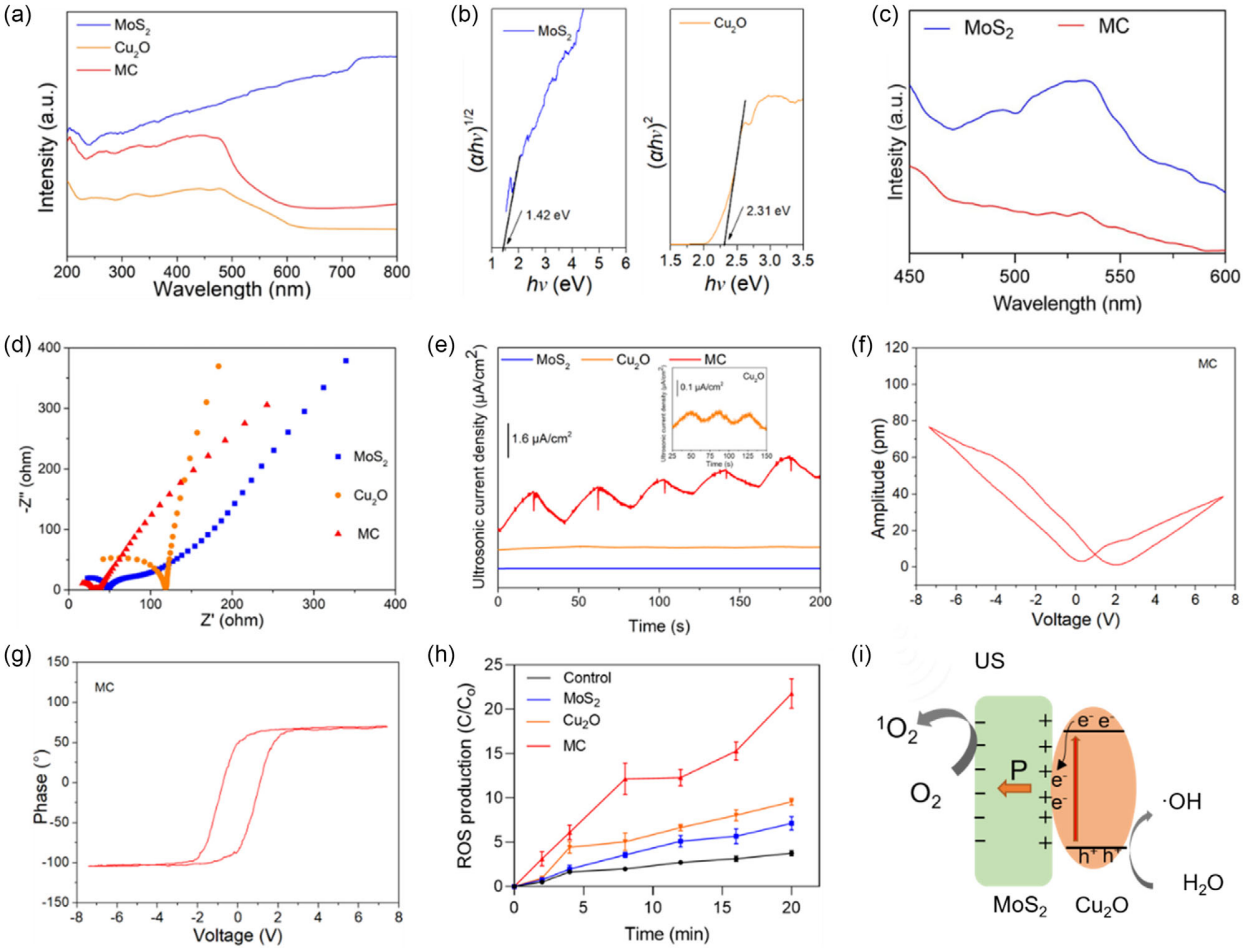
Piezocatalytic performance of different samples. a) The UV–visible absorption of different samples, b) the band structure calculated by UV–visible absorption spectrum of MoS_2_ and Cu_2_O, c) photoluminescence (PL) spectra of different samples, d) electrochemical impedance spectroscopy (EIS) spectra of samples under US irradiation, e) ultrasonic current density of the samples, f) piezoelectric force microscopy (PFM) amplitude of MC, g) PFM hysteresis loop plot of MC, and h) reactive oxygen species (ROS) production with 2′,7′‐dichlorofluorescein (DCFH) fluorescence probe under US irradiation for 20 min. i) Schematic illustration of the piezocatalytic mechanism.

The XPS results indicated a strong electron interaction and charge transfer between Cu_2_O and MoS_2_. Therefore, the sonodynamic effects of MC were measured by the following experiments. As shown in **Figure** **4**a, the UV–vis diffuse reflectance spectra of the different samples were obtained to measure changes in their optical properties. The MC showed large absorption compared with Cu_2_O, suggesting a higher tendency to be activated by exogenous stimuli with lower energy in MC. Figure 4b presents the bandgap values of the different materials calculated by UV–vis spectra (Figure 4a). The bandgap value of Cu_2_O was calculated to be 2.31 eV, and the bandgap value of MoS_2_ was calculated to be 1.42 eV. As shown in Figure S7, Supporting Information, the bandgap of MC is minimal compared to MoS_2_ and Cu_2_O. Furthermore, the photoluminescence (PL) spectra (Figure 4c) were used to measure the electron–hole combination efficiency of the various samples. Compared with MoS_2_, the MC showed lower intensity, indicating that the piezoelectric potential separating the electron–hole pair of the MC had the lowest recombination rate.^[^ ^41^ ^]^ Figure 4d shows the electrochemical impedance spectroscopy (EIS) curves of the as‐prepared samples. Compared with Cu_2_O and MoS_2_, MC showed the smallest electric impendence under US irradiation. The electron‐transfer resistance was reduced by the coupling of MoS_2_ and Cu_2_O, indicating that the interface of MC could favor charge transfer. Figure 4e shows the piezoelectric current densities of the as‐prepared samples under US (1.5 W cm^−2^, 1 MHz) irradiation. Among the three samples, MC demonstrated the strongest piezoelectric current density, followed by MoS_2_ and Cu_2_O. This indicates the best transfer ability of charge carriers of MC under US (1.5 W cm^−2^) irradiation. The results were in accordance with the EIS results (Figure 4d). Figure S8, Supporting Information, shows the current potential (current–voltage [*I–V*]) curves of the different samples under US (1.5 W cm^−2^) irradiation. The results show that MC has the most free electrons under US excitation, which also corresponds to the results of US current of Figure 4e. To further explore the catalytic mechanism, we performed piezoelectric force microscopy (PFM) to understand the piezoelectricity of MC. As shown in Figure 4f,g, a typical amplitude voltage butterfly loop and a phase change close to ≈180° were observed under the applied voltage of 8 eV, proving that MC possessed typical piezoelectric properties. Therefore, as a mechanical wave, US can stimulate MC to produce polarization (e.g., the piezoelectric effect). Furthermore, total ROS generation under US irradiation was measured using the dye 2′,7′‐dichlorofluorescein (DCFH) (Figure 4h). Under US (1.5 W cm^−2^) irradiation, the control group and MoS_2_ showed very little ROS. The ROS yields of the Cu_2_O group were slightly higher than those of the control group. In contrast, the combination between MoS_2_ and Cu_2_O induced the most yields of ROS from the synthesized MC under US treatment for 20 min, indicating the best US catalytic performance. In conclusion, the mechanism behind the sonocatalytic of MC is shown in Figure 4i. Given that the sheet of molybdenum sulfide had a good piezoelectric effect, the energy generated by US could excite molybdenum sulfide to produce a polarized charge.^[^ ^42^ ^]^ After the formation of the heterojunction, the electrons and hole teams generated by ultrasonic excitation of cuprous oxide are separated, the electrons flow to the surface of the heterojunction, and the polarized charge is trapped. This causes more holes and polarized electrons to participate in the generation of ROS, thereby producing more ROS.

### 2.3 In Vitro Sonodynamic Antibacterial Performance of MoS_2_/Cu_2_O

**Figure 5:**
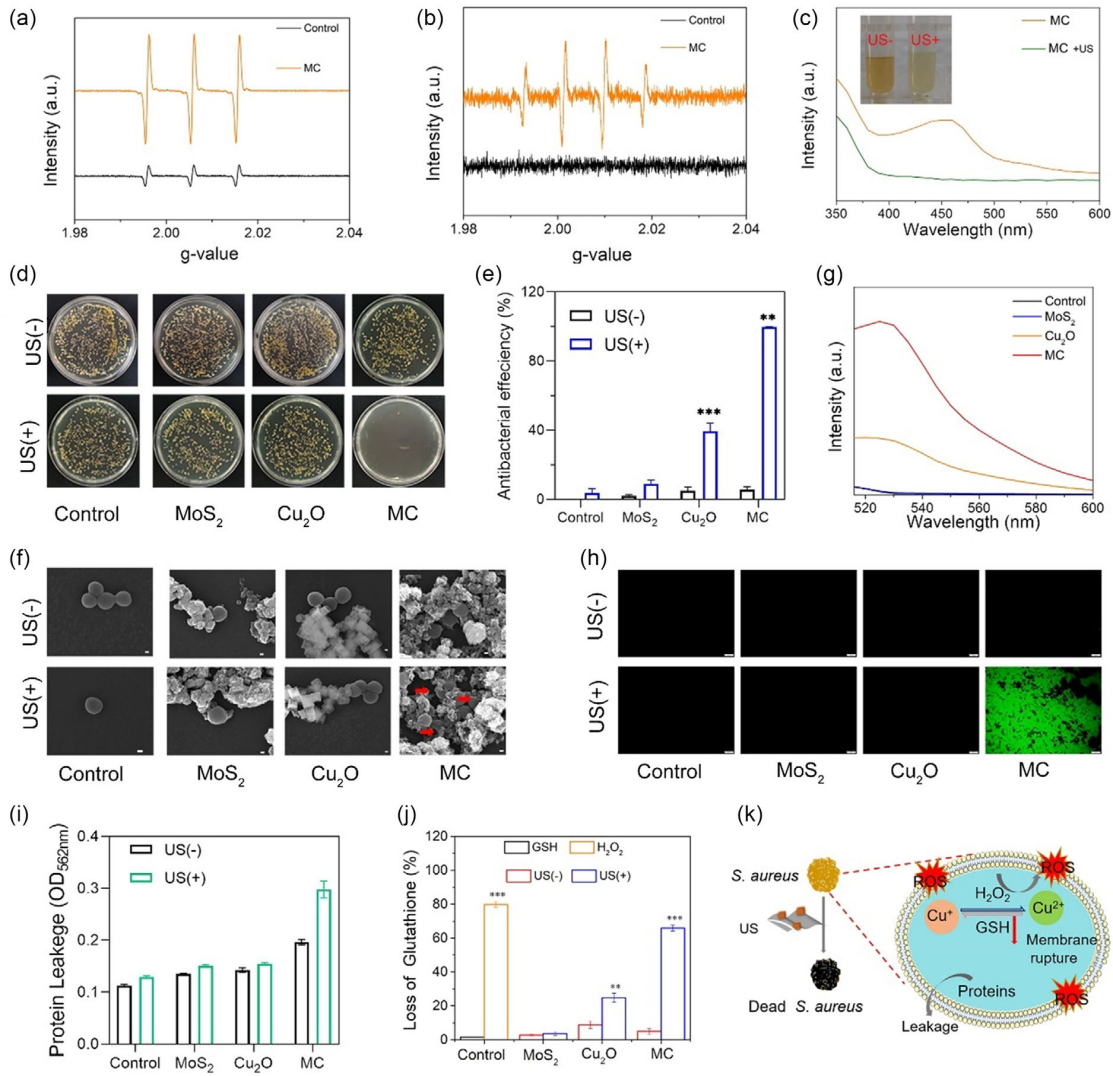
Sonocatalytic and antibacterial activity. a) ^1^O_2_ detection of MC and MC with US irradiation, b) ·OH detection of MC and MC with US irradiation. c) UV–vis absorbance spectra of neocuproine after different treatments. d) The spread plate images of *Staphylococcus aureus* of the different materials with and without US irradiation for 20 min. e) The antibacterial efficiency of different samples, according to the spread plate pictures (the error bars indicate means ± SD, *n* = 3. *p < 0.05, **p < 0.01, ***p < 0.001), and f) morphologies of *S. aureus* in different groups (scale bar = 500 nm). g) Fluorescence intensity curves of ROS in bacteria under ultrasound for different materials in figure (h). h) Intracellular ROS detection using DCFH‐DA of *S. aureus* and the corresponding fluorescent image. i) Detection of protein leakage of *S. aureus* under US irradiation through bicinchoninic acid assay (BCA). j) Glutathione (GSH) deletion ability of different samples. k) The antibacterial mechanism diagram.

Given the excellent ultrasonic response and piezoelectric catalytic ability of heterojunctions, we further explored the types of ROS under ultrasonic excitation. Electron‐spin resonance spectroscopy (**Figure** **5**a) was used to further verify the type of ROS produced by the as‐prepared samples under 1.5 W cm^−2^ US excitation. By using 2,2,6,6‐tetramethylpiperidine as a trapping agent, typical spectra of⋅^1^O2 radical were detected in each group with US (1.5 W cm^−2^) treatment. Compared with group MC, group MC + US showed the highest singlet oxygen signal. This indicates that MC can produce a large amount of ROS under US. As shown in Figure 5b, in the absence of ultrasonic excitation, there was no hydroxyl‐free signal generation in the heterojunction, whereas after ultrasonic excitation, the hydroxyl radical signal was significantly enhanced. Singlet oxygen and hydroxyl radicals produced by US excitation could effectively remove bacterial infections. To demonstrate whether US can induce the transition between Cu(I) and Cu(II), we used neocuproine as the detection reagent. When Cu^+^ is present, Cu^+^ will combine with neocuproine to form [Cu(neocuproine)_2_]^+^ to make the solution yellow, and when Cu^+^ decreases, the color of the solution decreases. Figure 5c shows that when the US is applied to MC, the color of the solution becomes lighter, and the absorption intensity at 450 nm decreases, indicating that Cu(I) has changed to Cu(II). Because of the piezoelectric action of MC, which causes an excess of ROS to be produced when US is excited, we employed the spread plate method to count the number of *S. aureus* colonies to assess the antibacterial performance of MC. Figure S9, Supporting Information, shows the antibacterial performance of MC at different concentrations, and after comparison, we determined that 500 ppm is the optimal antibacterial concentration for MC. As shown in Figure 5d, there was no significant reduction in colonies in all experimental groups without US excitation compared to the control group. On the contrary, under US excitation, the antibacterial efficiency of Cu_2_O group was 38.51% relative to the control group. This was due to the action of copper ions under US, which promotes the conversion of Cu(I) and Cu(II) in US.^[^ ^20^ ^]^ Although MoS_2_ exhibited piezoelectric performance under US excitation, due to the rapid recombination of polarized electrons and hole pairs, the ROS yield was low, so its antibacterial effect was not significant. In contrast, after US irradiation for 20 min, the antibacterial efficiency of MC was 99.85%, which was due to the synergistic effects of enhanced ultrasonic performance and Cu (II). Figure 5e shows a histogram of the antibacterial efficiency corresponding to Figure 5d. Figure 5f reveals the morphology of the bacteria, and the structure of the bacteria was relatively complete in each group without US irradiation. In contrast, the cell membranes of the bacteria were damaged to varying degrees after US irradiation for 20 min. Especially in the MC group, the cell membranes of the bacteria were severely folded, and the bacteria were deformed. The outcome corresponded well to the results shown in Figure 5d. To further investigate the antibacterial mechanism of MC under US irradiation, we conducted the following experiments.

A probe called 2′,7′‐dichlorofluorescein diacetate (DCFH‐DA) can identify intracellular ROS, which can penetrate the bacteria's interior and hydrolyze into DCFH, which then emits fluorescence. The more ROS within the bacteria, the brighter the fluorescence. Figure 5g shows the fluorescence intensity of intracellular ROS in each group under US at 525 nm. The results show that the MC group had the strongest intensity at 525 nm and produced the most intracellular ROS, which would cause severe oxidative stress in bacteria. The results of Figure 5h correspond well to those in Figure 5g.

As shown in Figure 5i, compared to the control group, the protein leakage from *S. aureus* was negligent in the MoS_2_ group, with or without US irradiation. In contrast, under US irradiation, the protein leakage of bacteria in the Cu_2_O group increased significantly. The protein leakage of bacteria with US (1.5 W cm^−2^) irradiation in the MC group was particularly obvious relative to that in the control group. As an endogenous antioxidant, GSH was used as an indicator of oxidative stress to assess the oxidative properties of all the groups.^[^ ^43^ ^]^ As shown in Figure 5j, compared with the control group, GSH depletion in the MoS_2_ group was negligible, with and without US excitation. In the absence of US, the depletion of Cu_2_O group compared with the control GSH was 8.81%, which was caused by the oxidation of Cu^+^ on the surface of Cu_2_O. The depletion of GSH in the Cu_2_O + US group was 24.75%, indicating that US could convert Cu^+^ in Cu_2_O to Cu^2+^ to participate in the depletion of GSH. In comparison, the MC + US group had the largest depletion of 65.89%, which showed that the MC could effectively oxidize GSH under US irradiation. Figure 5k shows the antibacterial mechanism of MC.

### 2.4 In Vitro Cytocompatibility

**Figure 6:**
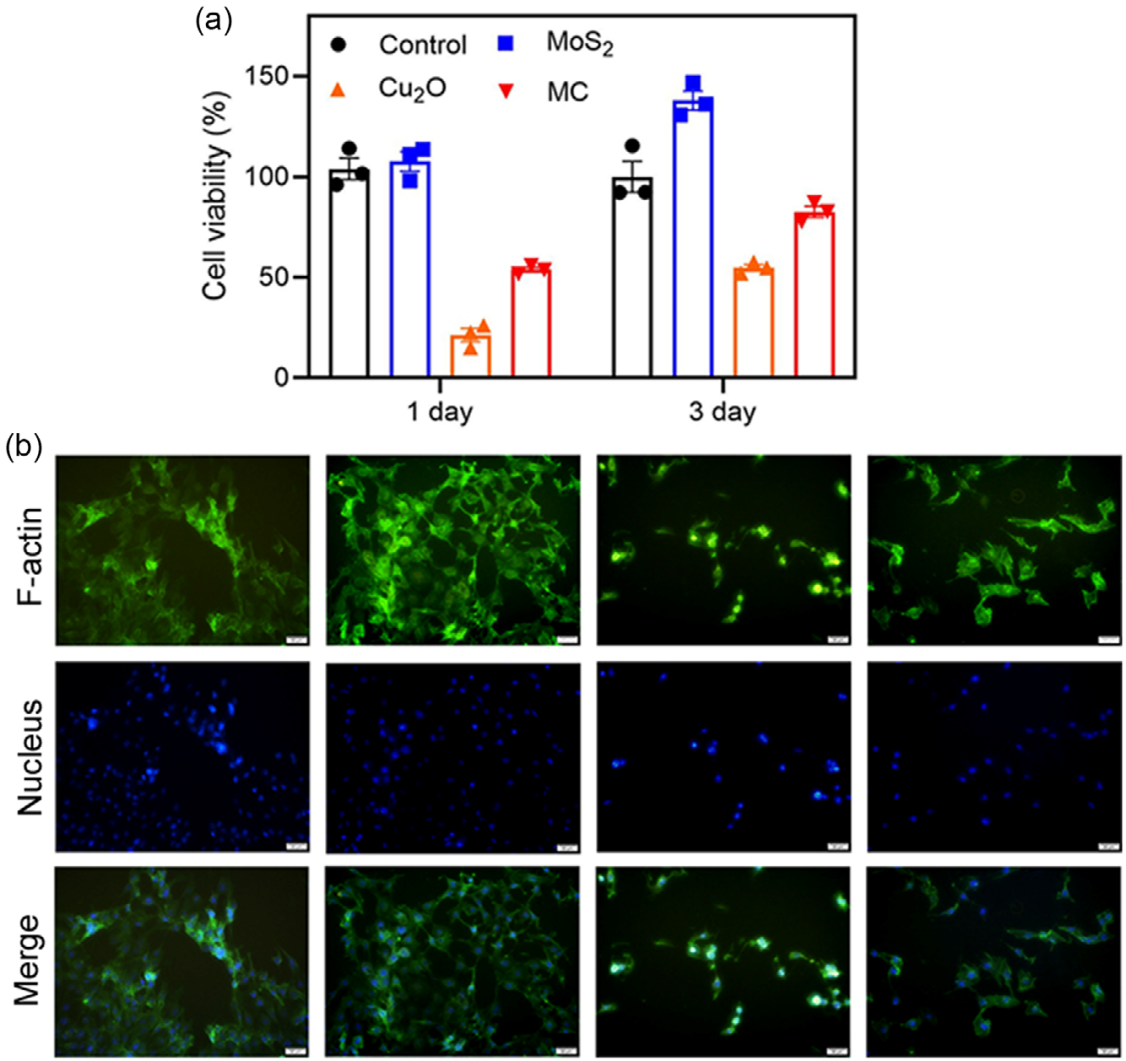
a) MTT cell viability of NIH3T3 cells cocultured with MoS_2_, Cu_2_O, and MC in dark condition (*n* = 3). b) Fluorescein isothiocyanate (FITC)/4,6‐diamino‐2‐phenyl indole (DAPI) fluorescence images of NIH‐3T3 cells cocultured with MoS_2_, Cu_2_O, and MC (scale bar, 50 μm).

NIH‐3T3 is crucial for wound healing. It was used to assess the biocompatibility of different materials. Using the 3‐(4,5‐dimethylthiazol‐2‐yl)‐2,5‐diphenyltetrazolium bromide (MTT) method, we investigated the cytocompatibility of the materials. As shown in **Figure** **6**a, after coculture of cells and materials for 1 day, the MoS_2_ group showed good biocompatibility, which might be ascribed to the nanosheet structure of MoS_2_ because this structure could provide sufficient active sites for the cell to proliferate and expand. The Cu_2_O group showed obvious toxic effects on the cells, which might be ascribed to excess Cu^+^.^[^ ^44^ ^]^ However, after 3 days of culture, the cell viability of MC increased up to over 80%, indicating that MC had good cytocompatibility. As shown in Figure 6b, the fluorescence staining images of the cells showed that the MoS_2_ group exhibited more filopodia extensions around compared to the control group. This result indicates that the nanosheet structure of MoS_2_ facilitated fibroblasts to migrate and proliferate. However, the large amount of Cu ions (Cu^+^, Cu^2+^) released induced the death of cells, which contributed to the shrinking in the shape of the cells in the Cu_2_O group. Furthermore, the cells showed filopodia extending around due to the good biocompatibility of MC. Figure 6b is in accordance with Figure 6a (MTT assay) and further demonstrates that MC had good cytocompatibility.

## 3 Discussion and Conclusion

In this work, we developed MC nanocomposites with piezoelectric and valence‐adjustable heterostructures for sonodynamic antibacterial therapy. On the one hand, the establishment of the heterojunction interface prevented the recombination of the piezoelectrically polarized electrons and holes generated by the US excitation of MoS_2_, which improved the sonocatalytic ability of MC. On the other hand, the conversion of Cu(I) and Cu(II) in Cu_2_O due to US propelling increased the yield of electrons, and the generated Cu(II) ions could oxidize bacterial endo‐GSH, thus reducing bacterial activity. Because of the high ROS production of MC, *S. aureus* could be effectively killed under ultrasonic excitation at 1.5 W cm^−2^ for 20 min. In vitro cytotoxicity tests revealed that MoS_2_@Cu_2_O had good cytocompatibility. The current study will provide new insights into developing not only piezoelectric US‐excited bacteria‐killing materials but also piezoelectric devices, such as piezoelectric pacemakers and piezoelectric skin.

## Conflict of Interest

The authors declare no conflict of interest.

## Supporting information

Supplementary Material

## Data Availability

The data that support the findings of this study are available in the supplementary material of this article.

## References

[1] R. S. Wallis , A. O’Garra , A. Sher , A. Wack , Nat. Rev. Immunol. 2023, 23, 121. 35672482 10.1038/s41577-022-00734-z PMC9171745

[2] S. S. Jean , Y. Chang , W. Lin , W. S. Lee , P. R. Hsueh , C. W. Hsu , J. Clin. Med. 2020, 9, 275. 31963877 10.3390/jcm9010275 PMC7019939

[3] V. Kumar , Front. Immunol. 2020, 11, 1722. 32849610 10.3389/fimmu.2020.01722 PMC7417316

[4] M. Taati Moghadam , A. Khoshbayan , Z. Chegini , I. Farahani , A. Shariati , Drug Des. Dev. Ther. 2020, 14, 1867. 10.2147/DDDT.S251171 PMC7237115 32523333

[5] C. Wang , Y. Luo , X. Liu , Z. Cui , Y. Zheng , Y. Liang , Z. Li , S. Zhu , J. Lei , X. Feng , S. Wu , Bioact. Mater. 2022, 13, 200. 35224302 10.1016/j.bioactmat.2021.10.033 PMC8843951

[6] T. M. Uddin , A. J. Chakraborty , A. Khusro , B. R. M. Zidan , S. Mitra , T. B. Emran , N. Koirala , J. Infect. Public Health 2021, 14, 1750. 34756812 10.1016/j.jiph.2021.10.020

[7] J. Min , K. Y. Choi , E. C. Dreaden , R. F. Padera , R. D. Braatz , M. Spector , P. T. Hammond , ACS Nano 2016, 10, 4441. 26923427 10.1021/acsnano.6b00087 PMC6501197

[8] Y. Li , X. Liu , L. Tan , Z. Cui , X. Yang , Y. Zheng , K. W. K. Yeung , P. K. Chu , S. Wu , Adv. Funct. Mater. 2018, 28, 1800299.

[9] X. Kong , X. Liu , Y. Zheng , P. K. Chu , Y. Zhang , S. Wu , Mater. Sci. Eng., R 2021, 145, 100610.

[10] T. Wei , Q. Yu , H. Chen , Adv. Healthcare Mater. 2019, 8, 1801381. 10.1002/adhm.201801381 30609261

[11] C. Wang , J. Li , X. Liu , Z. Cui , D. Chen , Z. Li , Y. Liang , S. Zhu , S. Wu , Biomater. Sci. 2020, 8, 4216. 32578588 10.1039/d0bm00872a

[12] X. Qian , Y. Zheng , Y. Chen , Adv. Mater. 2016, 28, 8097. 27384408 10.1002/adma.201602012

[13] W. Yue , L. Chen , L. Yu , B. Zhou , H. Yin , W. Ren , C. Liu , L. Guo , Y. Zhang , L. Sun , K. Zhang , Y. Chen , Nat. Commun. 2019, 10, 2025. 31048681 10.1038/s41467-019-09760-3 PMC6497709

[14] Y. He , J. Wan , Y. Yang , P. Yuan , C. Yang , Z. Wang , L. Zhang , Adv. Healthcare Mater. 2019, 8, 1801254. 10.1002/adhm.201801254 30844136

[15] H. Liu , J. Li , X. Liu , Z. Li , Y. Zhang , Y. Liang , Y. Zheng , S. Zhu , Z. Cui , S. Wu , ACS Nano 2021, 15, 18505. 34739223 10.1021/acsnano.1c08409

[16] J. Lei , C. Wang , X. Feng , L. Ma , X. Liu , Y. Luo , L. Tan , S. Wu , C. Yang , Chem. Eng. J. 2022, 435, 134624.

[17] Y. Zhao , T. Huang , X. Zhang , Y. Cui , L. Zhang , L. Li , BMEMat 2023, 1, e12006.

[18] Y. Zhao , S. Wang , Y. Ding , Z. Zhang , T. Huang , Y. Zhang , X. Wan , Z. Wang , L. Li , ACS Nano 2022, 16, 9304. 35699224 10.1021/acsnano.2c01968

[19] Z. Liu , S. Zhang , Y. Jin , H. Ouyang , Y. Zou , X. Wang , L. Xie , Z. Li , Sci. Technol. 2017, 32, 064004.

[20] S. Xu , L. Guo , Q. Sun , Z. Wang , Adv. Funct. Mater. 2019, 29, 1808737.

[21] Y. Su , L. Zhang , W. Wang , X. Li , Y. Zhang , D. Shao , J. Mater. Chem. A 2018, 6, 11909.

[22] X. Feng , J. Lei , L. Ma , Q. Ouyang , Y. Zeng , H. Liang , C. Lei , G. Li , L. Tan , X. Liu , C. Yang , Small 2022, 18, 210577. 10.1002/smll.202105775 34889522

[23] M. Wu , Z. Zhang , Z. Liu , J. Zhang , Y. Zhang , Y. Ding , T. Huang , D. Xiang , Z. Wang , Y. Dai , X. Wan , S. Wang , H. Qian , Q. Sun , L. Li , Nano Today 2021, 37, 10110.

[24] W. Wu , Z. Wang , Nat. Rev. Mater. 2016, 1, 16031.

[25] L. Banci , I. Bertini , S. Ciofi-Baffoni , T. Kozyreva , K. Zovo , P. Palumaa , Nature 2010, 465, 645. 20463663 10.1038/nature09018

[26] J. Ouyang , A. Xie , J. Zhou , R. Liu , N. Kong , Y. Sang , W. Tao , Chem. Soc. Rev. 2022, 51, 4996. 35616098 10.1039/d1cs01148k

[27] Y. Zhou , S. Fan , L. Feng , X. Huang , X. Chen , Adv. Mater. 2021, 33, e2104223. 34580933 10.1002/adma.202104223

[28] X. Qin , C. Wu , D. Niu , L. Qin , X. Wang , Q. Wang , Y. Li , Nat. Commun. 2021, 12, 5243. 34475406 10.1038/s41467-021-25561-z PMC8413279

[29] W. Feng , Z. Liu , L. Xia , M. Chen , X. Dai , H. Huang , C. Dong , Y. He , Y. Chen , Angew. Chem., Int. Ed. 2022, 61, e202212021. 10.1002/anie.202212021 36198660

[30] L. Zhang , H. Wang , ACS Nano 2011, 5, 3257. 21351790 10.1021/nn200386n

[31] J. Li , X. Jin , R. Li , Y. Zhao , X. Wang , X. Liu , H. Jiao , Appl. Catal., B. 2019, 240, 1.

[32] K. Sunada , M. Minoshima , K. Hashimoto , J. Hazard. Mater. 2012, 235, 265. 22902129 10.1016/j.jhazmat.2012.07.052

[33] Y. Pu , W. Lin , Y. J. Hsu , Appl. Catal., B 2015, 163, 343.

[34] W. Zhang , B. Wang , C. Hao , Y. Liang , H. Shi , L. Ao , W. Wang , J. Alloys Compd. 2016, 684, 445.

[35] M. Deng , Z. Li , X. Rong , Y. Luo , B. Li , L. Zheng , X. Wang , F. Lin , A. J. Meixner , K. Braun , X. Zhu , Z. Fang , Small 2020, 16, 2003539. 10.1002/smll.202003539 32964680

[36] P. Liu , J. Zhu , J. Zhang , K. Tao , D. Gao , P. Xi , Electrochim. Acta 2018, 260, 24.

[37] E. Yu , H. C. Kim , H. J. Kim , S. Y. Jung , K. S. Ryu , S. I. Choi , J. W. Hong , Appl. Surf. Sci. 2021, 538, 148159.

[38] Y. Yuan , W. Wang , Y. Shi , L. Song , C. Ma , Y. Hu , J. Hazard. Mater. 2020, 382, 121028. 31473517 10.1016/j.jhazmat.2019.121028

[39] G. Yilmaz , T. Yang , Y. Du , X. Yu , Y. P. Feng , L. Shen , G. W. Ho , Adv. Sci. 2019, 6, 1900140. 10.1002/advs.201900140 PMC6685470 31406663

[40] Y. Yao , H. Hu , H. Zheng , F. Wei , M. Gao , Y. Zhang , S. Wang , Chem. Eng. J. 2020, 398, 125455.

[41] Z. Yang , C. Chen , B. Li , Y. Zheng , X. Liu , J. Shen , Y. Zhang , S. Wu , Chem. Eng. J. 2023, 451, 139127.

[42] J. Li , X. Liu , Y. Zheng , Z. Cui , H. Jiang , Z. Li , L. Zhu , S. Wu , Adv. Mater. 2023, 2210296. 10.1002/adma.202210296 36626342

[43] L. H. Fu , Y. Wan , C. Qi , J. He , C. Li , C. Yang , H. Xu , J. Lin , P. Huang , Adv. Mater. 2021, 33, 2006892. 10.1002/adma.202006892 33394515

[44] Q. Yang , Y. E. Wang , Q. Yang , Y. Gao , X. Duan , Q. Fu , C. Chu , X. Pan , Y. Sun , Biomaterials 2017, 146, 72. 28898759 10.1016/j.biomaterials.2017.09.008

