# Supplementary material for: Ultrasound‐Activated Piezoelectric MoS2 Enhances Sonodynamic for Bacterial Killing
Source: Small Sci. 2023 Apr 19;3(7):2300022. doi: 10.1002/smsc.202300022 (PMC11935888; doi:10.1002/smsc.202300022)
Supplement: Supplementary file 1 — Supplementary Material [file SMSC-3-2300022-s001.pdf]

Supporting Information

Ultrasound-activated piezoelectric MoS_2 enhances sonodynamic for bacterial killing

Chaofeng Wang, Wenchan sun, Yiming Xiang, Shuilin Wu, Yufeng Zheng, Yu Zhang[d], Jie Shen *, Lei Yang, Chunyong Liang, Xiangmei Liu**

C. Wang, Prof. L. Yang, Prof. C. Liang, Prof. X. Liu, School of Life Science and Health Engineering, Hebei University of Technology, Xiping Avenue 5340, Tianjin 300401, China.
E-mail: xm.liu@hubu.edu.cn; (X. Liu)

W. Sun, Y. Xiang, Prof. X. Liu, Biomedical Materials Engineering Research Center, Hubei Key Laboratory of Polymer Materials, Ministry-of-Education Key Laboratory for the Green Preparation and Application of Functional Materials, School of Materials Science & Engineering, Hubei University, Wuhan 430062, China.
E-mail: xm.liu@hubu.edu.cn (X. M. Liu)

Prof. Y. Zheng, Prof. S. Wu, School of Materials Science & Engineering, Peking University, Yiheyuan Road 5#, Beijing 100871, China
E-mail: slwu@pku.edu.cn (S. Wu)

Prof. Y. Zhang, Department of Orthopedics, Guangdong Provincial People's Hospital, Guangdong Academy of Medical Sciences, Zhongshan 2nd Road 106#, Guangzhou 510080, China

Prof. J. Shen, Shenzhen Key Laboratory of Spine Surgery, Department of Spine Surgery, Peking University Shenzhen Hospital, Lianhua Road 1120#, Futian District, Shenzhen, China
E-mail: jayjayson909@gmail.com (J. Shen)

1. Experimental section

1.1. Materials preparation

First, mix the sodium molybdate dihydrate (1.693 g) with deionized (40 mL) under vigorous stirring, and then take the thiourea (2.664 g) into the solution. After 30 min, the pH of the solution was brought down to less than 1.0 using HCl (2M). After thoroughly mixing, the solution was transferred to a 100 mL Teflon-lined stainless steel, and it heat up to 200 ^{o}C at 5 ^{o}C/min for 24 hours. Following natural cooling, the sample was washed by distilled water and ethanol by centrifugation at 9000 r/min, and then dried in an oven at 60 ^{o}C for 4 h.

To prepare MoS_2/Cu_2O (MC), the $CuCl_2$ of 0.1 M was added to 500 mL of distilled water, and the mixture was vigorously agitated at bath temperature of 55 °C for 30 min. Second, 30 mg of MoS_2 was added, and the mixture was rapidly agitated for 30 minutes. After that, NaOH (2M, 50mL) was added to the mixed solution under vigorous stirring. And then ascorbic acid (0.6 M, 50 mL) was gradually added to the above solutions and then stirred for 5h. The solution was washed by centrifugation (7000 rpm) with water and ethanol several times. The obtained products were dried in vacuum at 60 °C for 6 h. Cu_2O can be obtained by removing MoS_2 in the above synthesis process.

1.2. characterization of MoS_2/Cu_2O (MC)

The microstructures and morphologies of synthesized MC were examined by field emission scanning electron microscope FE-SEM JSM7100F, JEOL, JP) and transmission electron microscope (TEM, TF20, FEI, USA). The phase structure and chemical composition of different samples was measured by X-ray diffraction (XRD, D8A25, Bruker, Germany) and x-ray photoelectron spectroscopy (Thermo Fisher Scientific 250Xi), respectively. Ultraviolet–visible (UV–vis) diffuse reflection spectrum (DRS) was detected by ultraviolet–visible (UV–vis) spectrophotometer (UV-3600, Shimadu, JP). Room temperature photoluminescence (PL) measurements were measured by a fluorescence spectrometer (LS-55, PE, USA). Ultrasound experiments of different samples were performed through an Intelect Mobile ultrasound (Chattanooga 2776, DJO Group, USA).

1.3. Electrochemistry measurements

The ultrasound electrochemical measurements of the samples (MoS_2, Cu_2O, MoS_2/Cu_2O) were taken through a three-electrode electrochemical workstation (CHI660E, China), wherein platinum was used as the counter electrode and Ag/AgCl electrode served as the reference electrode. Mix 1 mL of distilled water, 3 mg samples and 50 μL Nafion thoroughly, then drop onto the ITO, dry, and obtain a working electrode. $NaSO_4$ (0.5M) solution was used as the electrolyte. Ultrasonic apparatus with 1.5 W cm^{-2} was utilized as excitation source for photocurrent testing, electrochemical impedance spectroscopy (EIS) measurement and

1.4. Sonodynamic experiment

The ROS production of the samples (MoS_2, Cu_2O, MC) was detected by 2′,7′-dichlorodihydrofluorescein diacetate (DCFH-DA) under US irradiation (1.5 W cm^{-2}, 50% duty cycle, 1 MHz, 15 min). Briefly, add 100 μL of DCFH and 100 μL sample (0.4 mg/mL) and stimulated by US. The yields of ROS were checked every 5 min at 525 nm with excitation at 485 nm by a microplate reader (SpectraMax i3, Molecular Devices, USA). EPR (Bruker EMXplus) spectroscopy was used further detected the production of $^1O_2$. MC aqueous solution and 2,2,6,6-tetramethylpiperidine (TEMP) reagent were prepared. The MC solution was mixed with the diluted TEMP reagent, Ultrasound for 5 min, and the $^1O_2$ was measured by ESR spectrum.

1.5. *In Vitro* Antibacterial Properties

The antibacterial effect of the materials against Gram-positive Staphylococcus aureus (S. *aureus*) (ATCC 25923) (1×10^7 CFU/mL) was verified using a standard plate coating method. Briefly, 100 μL of bacterial suspension and 100 μL of 1 mg/mL of different samples (MoS_2, Cu_2O, MC) were placed in centrifuge tubes. And then, treat the bacterium-sample mixed suspension with 1.5 W/cm^2 US for 20 min. After that, the bacterial solution was sucked out and diluted 100 times with sterile LB medium. Spread 20 μL of bacterial solution evenly on agar plates and culture at 37 ^oC for 24 h. The bacterial colony number (C) was counted, and the antibacterial rate was calculated using the following equation: antibacterial ratio (%) = [C(control) − C(experiment group)]/C(control) × 100 %.

1.6. Bacterial Morphology

The treated bacteria were fixed with glutaraldehyde (2.5%) and static for two hours, the upper layer solution was removed, and the bacteria were then washed three times with PBS. Finally, The bacterial were then dehydrated for 15 minutes using a gradient alcohol (10%, 30%, 50%, 70%, 90%, 100%), respectively. The morphologies of bacteria were observed by FE-SEM.

1.7. BCA test

According to the manufacturer's nstructions, the BCA kit was used to measure the bacterial cell membrane damage in *S. aureus*. Briefly, the different samples (Control, MoS_2, Cu_2O, and MC) carried out the antibacterial experiment in accordance with the aforementioned antibacterial processes. After that, these suspensions are then centrifuged at 10,000 rpm for 5 min at 4 ºC. Add 25 μL of supernatant to 200 μL of BCA diluent and incubated for 15–30 min at 37 °C. Protein leakage is measured by testing the absorption value of the mixed solution at a microplate reader at 562 nm.

1.8. Ellman's assay

Place 200 μL of GSH (0.8×10^{-3} M) solution in a centrifuge tube, then add 100 μL of samples (PBS, MoS_2, Cu_2O, and MC) and mix well. The different groups were irradiated by US (20 min, 1.5 W cm^{-2}) or treated in the dark. 30% of H_2O_2 was used as a positive control group in the above experiment. Then 400 μL of Tris-HCl (0.05 M, pH = 8.0) and 100 μL of 5,5′-dithio-bis-(2-nitrobenzoic acid) (DTNB) (10 × 10-3 M) were added to the centrifuge tube and shook for 30 min. Finally, the optical density (OD) of the mixed solution at 410 nm was read by microplate reader.

1.9. Detection of intrabacterial ROS

The bacterial solution (1 mL of 10^9 CFU/mL) was centrifuged (5000 rpm for 6 min) and washed 3 times with PBS, then 100 μL of DCFH-DA diluted with PBS (1:2000) was added and incubated for 30 min at 37 ºC. After that, DCFH-DA was removed and the sample solution (500 μg/mL) was added, the mixture was illuminated with US for 20 min. And then absorption spectrum was detected at 525 nm, the bacteria was observed through a fluorescence microscope (IX73, Olympus, USA).

1.10. Cytocompatibility evaluation

The NIH-3T3 cells were used to the target cells to determine the cytotoxicity of the samples using 3-[4,5-dimethylthiazol-2-yl]-2,5-diphenyl etrazoliumbromide (MTT). After the NIH-3T3 cells were cultured to good condition, the solution was removed, and then new cell culture medium and sterilized materials (MoS_2, Cu_2O, MoS_2/Cu_2O) were added to different well plates in sequence. After 1 day and 3 days of incubation at 37 ^oC, remove the solution inside the plate, then add 200 μL of MTT (0.5mg/mL) solution to each well separately, and incubate at 37 °C for 4 h. After that, the MTT and add 200 μL of dimethyl sulfoxide (DMSO) were placed in each well at a time and shake for 15 min. To eliminate the influence of materials on the test, liquid centrifugation (6,000 rpm) for each group, and then take the upper layer overnight and read the OD values at 490 nm and 570 nm through SpectraMax I3 Molecular device.

After co-culture the material and NIH-3T3 cells for 1 day, discard the liquid in the plate and subsequently wash three times with PBS. After fixing 4% formaldehyde for 10 min, wash 3 times with PBS. The cytoplasm was dyed with FITC solution for 30 min. After cleaned with sterile PBS, the nucleus was dyed with DAPI solution for 30 s and washed with PBS for 3 times. Finally, the images obtained by inverted fluorescence microscope (IFM, Olympus, IX73)

1.11. Statistics

In order to ensure the scientificity and accuracy of the experimental datas and results, the statistical significance against all the experiments data was evaluated and analyzed by a previous way.

2. Supporting Figures

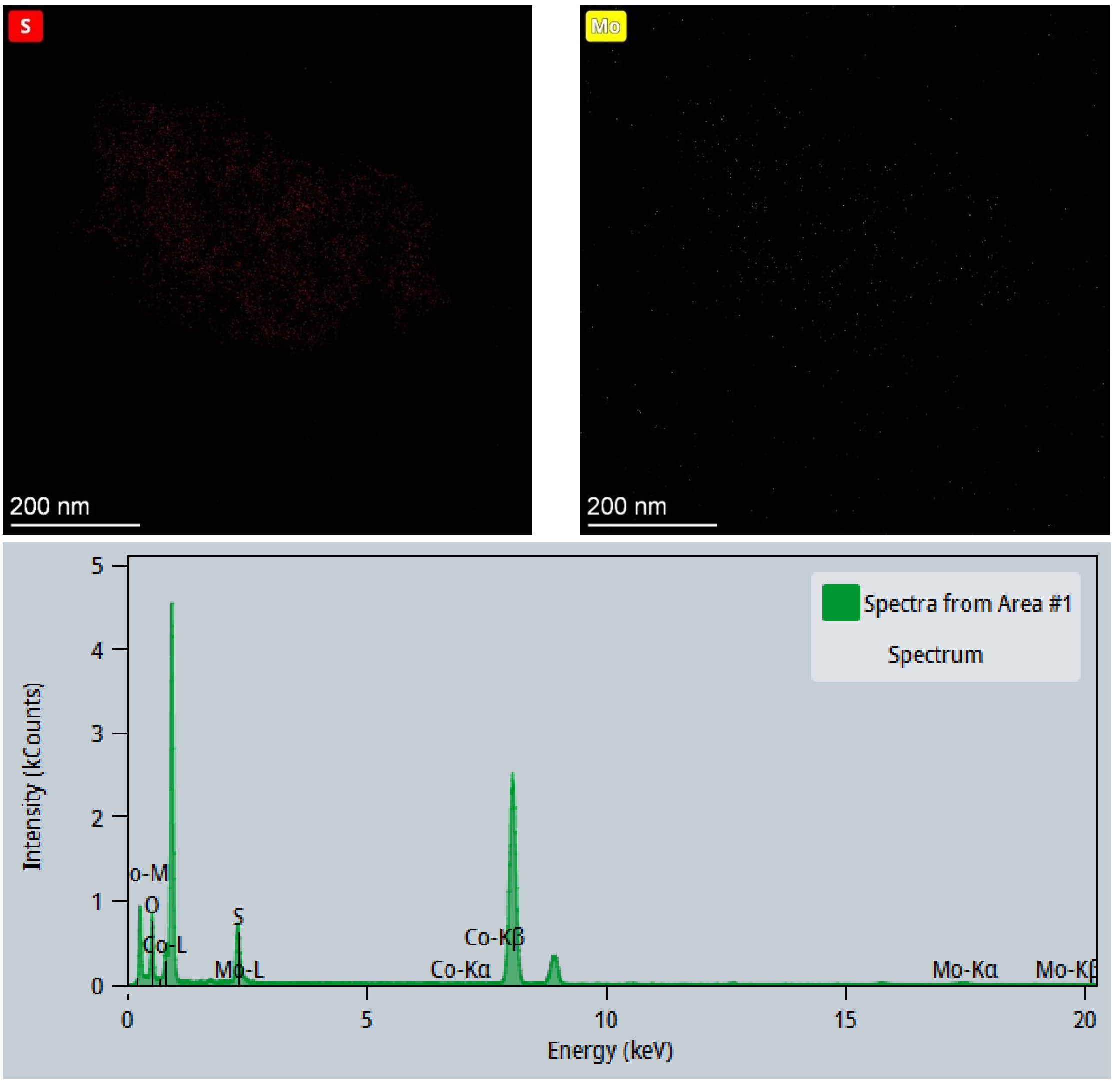

Figure S1. Elemental mapping of MoS_2 of (a) S, (b) Mo. (c) EDS detection corresponding to Figure 1c.

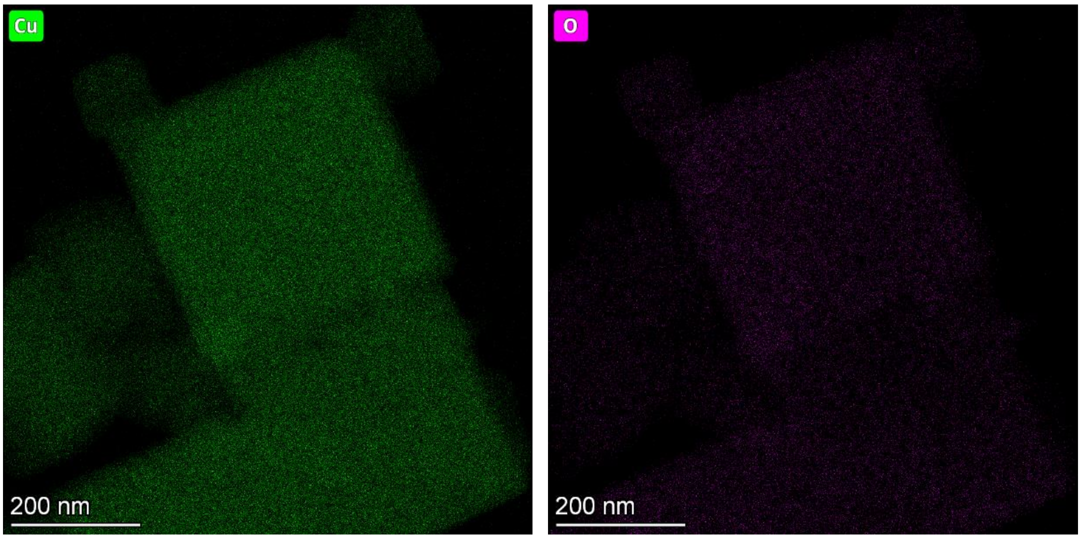

Figure S2. Elemental mapping of Cu_2O of (a)Cu, (b)O.

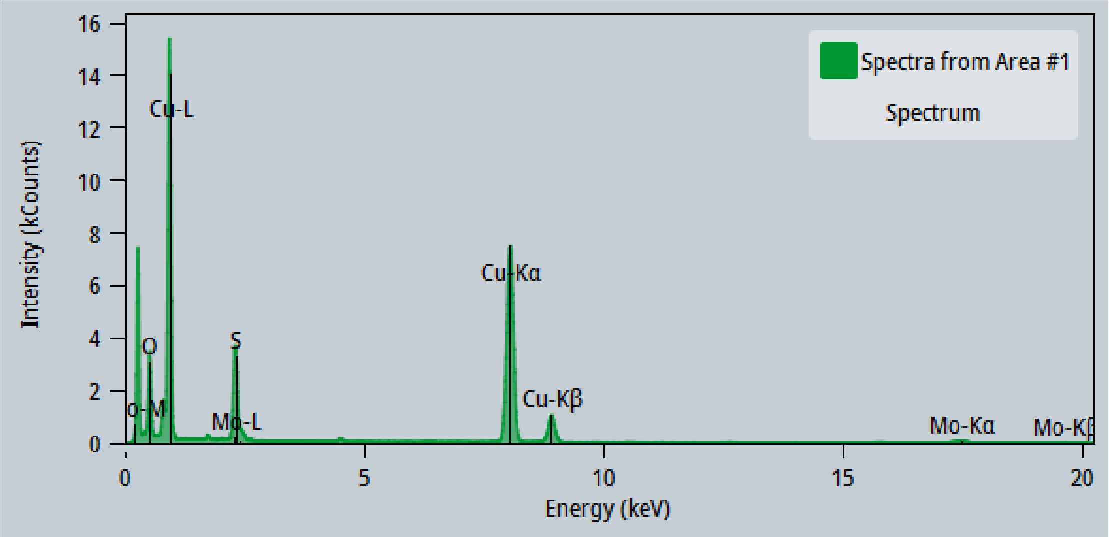

Figure S3. EDS detection corresponding to Figure 1i**.**

Elements	element content C_x (mg/kg)	element content W (%)
Mo	207884.64	20. 7884
Cu	597276.98	59.7277

Table 1. The elements content of Mo and Cu in MC

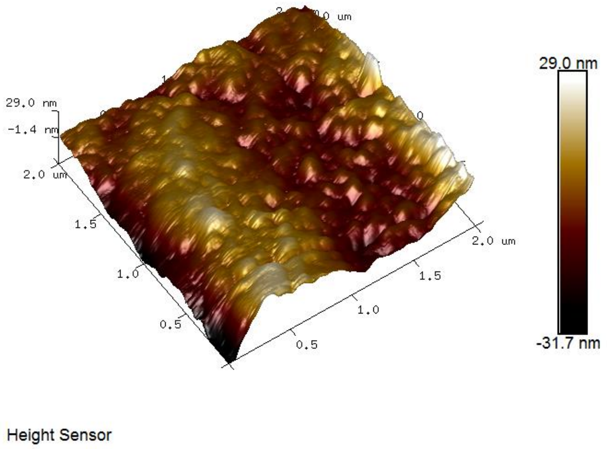

Figure S4. 3D section analysis of Cu_2O samples with AFM image.

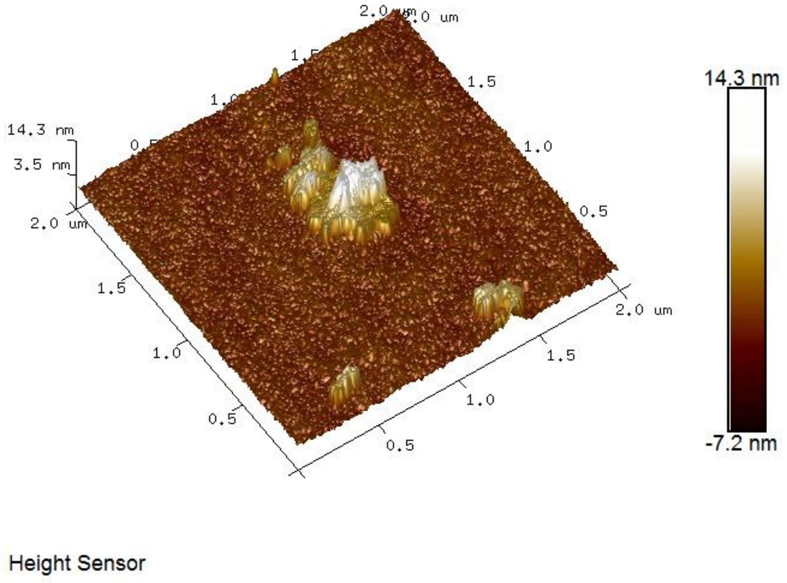

Figure S5. 3D section analysis of MoS_2 samples with AFM image.

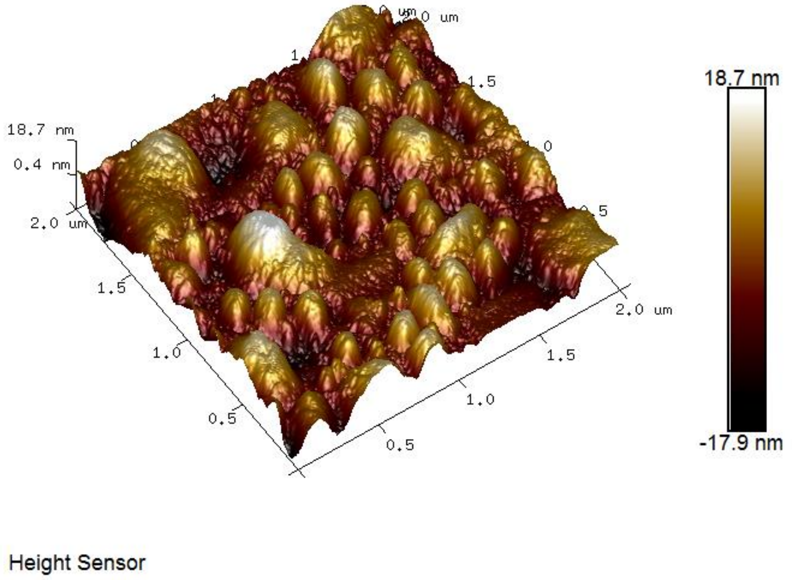

Figure S6. 3D section analysis of MC samples with AFM image.

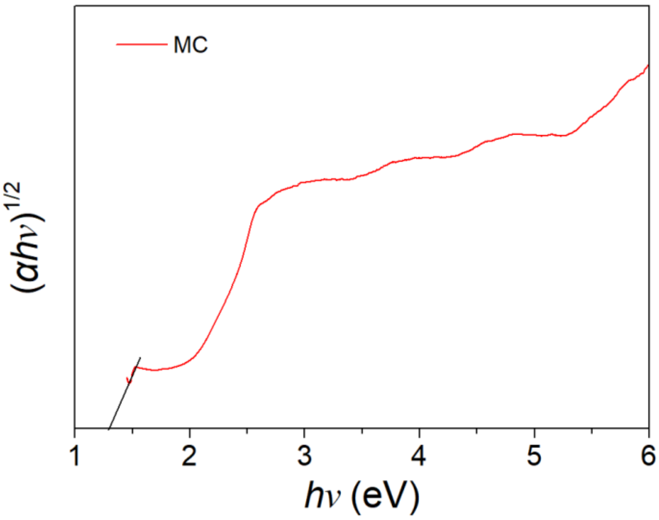

Figure S7. the band structure calculated by UV–visible absorption spectrum of MC.

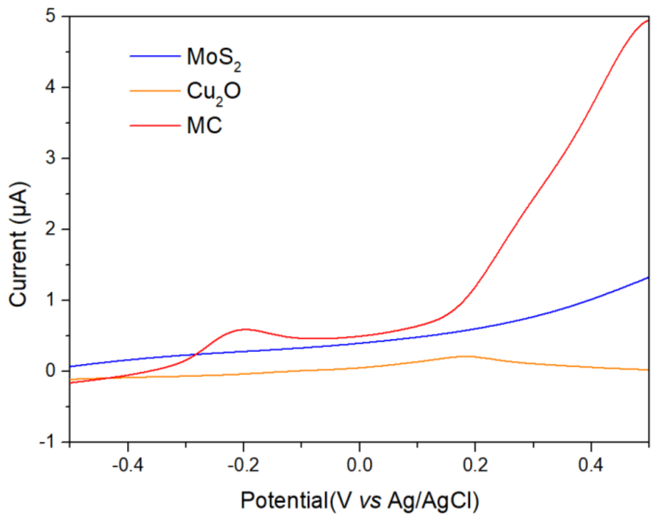

Figure S8. *I-V* curves of the MoS_2, Cu_2O and MC.

MC

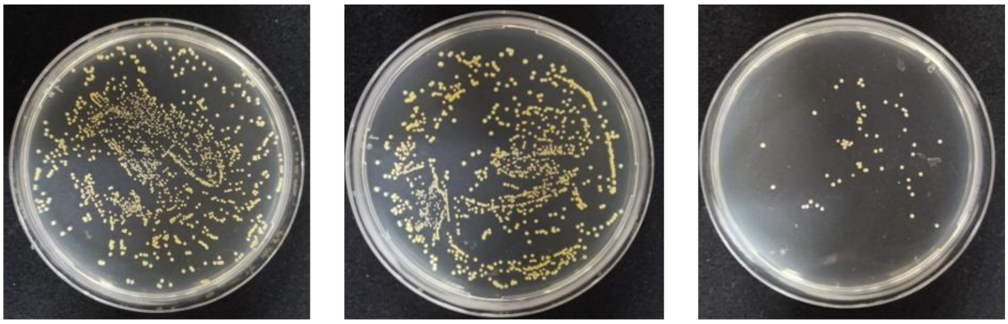

200 ppm | 400 ppm | 500 ppm

Figure S9. The spread plate images of *S. aureus* in different concentration MC.
